# Supplementary material for: Neuroimaging of intimate partner violence can underrepresent the most affected survivors
Source: bioRxiv. 2026 Sep 29:2026.09.23.753381. Preprint. [Version 1] doi: 10.64898/2026.09.23.753381 (PMC13648376; doi:10.64898/2026.09.23.753381)
Supplement: Supplementary file 1 [file NIHPP2026.09.23.753381v1-supplement-1.pdf]

bioRxiv preprint doi: https://doi.org/10.64898/2026.09.23.753381; this version posted September 29, 2026. The copyright holder for this preprint (which was not certified by peer review) is the author/funder, who has granted bioRxiv a license to display the preprint in perpetuity. It is made available under a CC-BY 4.0 International license.

Supplementary Information

Supplementary Table 1. Women with resting-state fMRI data had more education, higher employment, and lower psychiatric burden than women without imaging data. To determine whether the imaging sample differed from the broader enrolled cohort, we compared 61 women with resting-state fMRI data and 146 without these data across socioeconomic, psychiatric, neurobehavioral, injury, abuse, and demographic measures. Values are means (SD) unless otherwise stated. Strongly right-skewed count variables are summarized with medians [IQR] and Cliff's delta (δ), and the winsorized injury count is provided for comparison. Hedges *g* represents the standardized mean difference with percentile bootstrap 95% confidence intervals. Binary characteristics are summarized with Haldane-Anscombe-corrected odds ratios and 95% confidence intervals. The analyzed *n* is reported for each estimate and varies with missingness.

Characteristic	Imaged	Not imaged	Effect size [95% CI]	*n*
Education (years)	16.6 (2.6)	15.4 (2.9)	0.42 [0.13, 0.72]	61/146
Currently employed	75.4% (46/61)	59.3% (86/145)	OR 2.06 [1.06, 4.00]	206
Depression, PHQ-9	8.1 (5.2)	10.2 (5.9)	−0.37 [−0.64, −0.09]	61/146
Anxiety, GAD-7	6.9 (5.2)	8.9 (5.9)	−0.35 [−0.64, −0.07]	61/146
Post-traumatic stress, PCL-5	29.2 (17.4)	35.3 (19.9)	−0.32 [−0.59, −0.02]	61/146
Neurobehavioral symptoms, NSI total	20.3 (12.6)	27.4 (14.9)	−0.50 [−0.78, −0.21]	61/145
NSI vestibular	0.6 (0.6)	0.9 (0.9)	−0.38 [−0.63, −0.11]	61/145
NSI somatosensory	0.6 (0.5)	0.9 (0.7)	−0.41 [−0.65, −0.15]	61/145
NSI cognitive	0.9 (0.7)	1.2 (0.8)	−0.32 [−0.61, −0.03]	61/145
NSI affective	1.3 (0.8)	1.7 (0.9)	−0.46 [−0.76, −0.17]	61/145
Ongoing abuse	9.6% (5/52)	6.3% (5/79)	OR 1.57 [0.46, 5.40]	131
Raw partner-inflicted BI count median [IQR]	1 [0–5]	1 [0–4]	δ 0.03	61/146
Winsorized at 25 partner-inflicted BI count mean (SD)	4.5 (7.1)	4.2 (7.3)	0.03 [−0.25, 0.34]	61/146
Strangulation-related BI count	0 [0–0]	0 [0–1]	δ −0.04	61/146
Childhood maltreatment, CTQ	56.2 (21.9)	59.2 (22.1)	−0.14 [−0.44, 0.17]	61/146
Abuse severity, CASR-SF (past 12 months)	6.5 (11.1)	7.0 (11.1)	−0.04 [−0.33, 0.27]	61/146
Years since last IPV brain injury	10.9 (9.0)	10.9 (10.8)	0.00 [−0.36, 0.38]	37/85
Non-White race	25.0% (15/60)	31.7% (46/145)	OR 0.73 [0.37, 1.43]	205
Age (years)	40.2 (13.3)	41.4 (13.1)	−0.08 [−0.40, 0.21]	61/146
Highest symptom-burden quartile	11.5% (7/61)	30.8% (45/146)	OR 0.31 [0.13, 0.71]	207

bioRxiv preprint doi: https://doi.org/10.64898/2026.09.23.753381; this version posted September 29, 2026. The copyright holder for this preprint (which was not certified by peer review) is the author/funder, who has granted bioRxiv a license to display the preprint in perpetuity. It is made available under a CC-BY 4.0 International license.

Supplementary Table 2. Imaging criteria excluded three in five behaviorally eligible women. To identify the criteria that most often limited imaging eligibility, we summarized every recorded reason for neuroimaging ineligibility among 211 behaviorally eligible women. Women could meet more than one criterion, so percentages exceed 100% in total. The MRI contraindication item was unassessed in 58 women, producing different percentages for the full and assessed samples. Among ineligible women, 161 (76%) failed at least one criterion that remained in use, and 49 (23%) failed only criteria that were later removed. One participant was excluded for reasons noted during the interview, in which they discussed discomfort with MRI after a prior bad MRI experience.

Criterion	*n*	% of excluded	% of those assessed	Assessed
Criteria applied throughout				
Geographic location	64	30.3%	33.7%	190/211
Magnetic-resonance contraindication	58	27.5%	37.9%	153/211
Schizophrenia, bipolar disorder or autism	34	16.1%	16.3%	209/211
Moderate or severe traumatic brain injury	30	14.2%	14.6%	206/211
Confounding neurological disorder	29	13.7%	14.4%	201/211
Substance use history	28	13.3%	13.6%	206/211
Brain injury within three months	17	8.1%	8.2%	207/211
Pregnancy	1	0.5%	0.5%	197/211
Prior bad MRI experience	1	–	–	–
Criteria since relaxed or dropped				
Psychotropic-medication use	105	49.8%	51.5%	204/211
One or two partner-inflicted brain injuries	58	27.5%	29.1%	199/211
Age above the ceiling of 55 years	38	18.0%	18.5%	205/211

Supplementary Table 3. Motion quality control disproportionately excluded women with ten or more partner-inflicted BIs across all evaluated mean FD thresholds. To determine whether this association depended on the mean FD threshold, we compared exclusion among women reporting ten or more BIs and women reporting fewer BIs at 0.15, 0.20, and 0.30 mm. Values are the number and percentage excluded within each injury group among 61 women with rsfMRI data. The 0.20 mm threshold was primary, and two-sided Fisher exact *P* values are reported.

Threshold	Ten or more injuries excluded	Fewer than ten excluded	Fisher *P*
Mean FD ≥ 0.15 mm	6/11 (55%)	9/50 (18%)	.019
Mean FD ≥ 0.20 mm	4/11 (36%)	2/50 (4%)	.008
Mean FD ≥ 0.30 mm	2/11 (18%)	0/50 (0%)	.030

Supplementary Table 4. At the 0.30 mm mean FD threshold, the only women excluded reported ten or more partner-inflicted BIs. To describe how each quality-control threshold changed the excluded sample, we report the number and percentage excluded and the median BI count among excluded and retained women at 0.15, 0.20, and 0.30 mm. At the primary 0.20 mm threshold, the six excluded women reported 0, 0, 10, 13, 40, and 420 partner-inflicted BIs.

Threshold	*n* excluded	% of sample	Median injuries, excluded	Median injuries, retained
Mean FD ≥ 0.15 mm	15	24.6%	5.0	1.0

bioRxiv preprint doi: https://doi.org/10.64898/2026.09.23.753381; this version posted September 29, 2026. The copyright holder for this preprint (which was not certified by peer review) is the author/funder, who has granted bioRxiv a license to display the preprint in perpetuity. It is made available under a CC-BY 4.0 International license.

**Threshold**	***n* excluded**	**% of sample**	**Median injuries, excluded**	**Median injuries, retained**
Mean FD ≥ 0.20 mm	6	9.8%	11.5	1.0
Mean FD ≥ 0.30 mm	2	3.3%	11.5	1.0

Supplementary Table 5. The association between motion exclusion and ten or more BIs persisted after each excluded woman was removed in turn. To assess whether the result depended on the injury threshold or any one excluded observation, we repeated the primary 0.20 mm comparison at thresholds of any, five, and ten BIs and then removed each excluded woman in turn. Each row reports exclusion counts, an odds ratio with a 95% confidence interval, and a two-sided Fisher exact *P* value. Removing any one excluded woman with ten or more BIs changed P from .008 to .029. Mean FD was unrelated to continuous injury count, with Spearman ρ = 0.01, *P* = .918, and Pearson *r* = 0.26 for log-transformed injury count.

**Analysis**	**Exposed group excluded**	**Comparison excluded**	**Odds ratio [95% CI]**	**Fisher *P***
Ten or more vs fewer injuries	4/11 (36%)	2/50 (4%)	11.64 [2.07, 65.45]	.008
Five or more vs fewer injuries	4/17 (24%)	2/44 (5%)	5.67 [1.07, 29.89]	.046
Any injury vs none	4/41 (10%)	2/20 (10%)	0.89 [0.17, 4.59]	1.000
Leave-one-out 1: excluded participant with 0 injuries removed	4/11 (36%)	1/49 (2%)	19.40 [2.62, 143.57]	.003
Leave-one-out 2: excluded participant with 0 injuries removed	4/11 (36%)	1/49 (2%)	19.40 [2.62, 143.57]	.003
Leave-one-out 3: excluded participant with 40 injuries removed	3/10 (30%)	2/50 (4%)	9.05 [1.50, 54.55]	.029
Leave-one-out 4: excluded participant with 420 injuries removed	3/10 (30%)	2/50 (4%)	9.05 [1.50, 54.55]	.029
Leave-one-out 5: excluded participant with 10 injuries removed	3/10 (30%)	2/50 (4%)	9.05 [1.50, 54.55]	.029
Leave-one-out 6: excluded participant with 13 injuries removed	3/10 (30%)	2/50 (4%)	9.05 [1.50, 54.55]	.029
